# Controlling Non-Grain Production Based on Cultivated Land Multifunction Assessment

**DOI:** 10.3390/ijerph19031027

**Published:** 2022-01-18

**Authors:** Yue Su, Chong Su, Yan Xie, Tan Li, Yongjun Li, Yuanyuan Sun

**Affiliations:** 1 College of Economics & Management, Anhui Agricultural University, Hefei 230036, China; ysu@ahau.edu.cn (Y.S.); moreY@stu.ahau.edu.cn (Y.X.); litan@ahau.edu.cn (T.L.); 2 Institute of Agriculture Remote Sensing and Information Technology, College of Environmental and Resource Sciences, Zhejiang University, Hangzhou 310058, China; suchong@zju.edu.cn; 3 College of Big Data, Qingdao University of Science and Technology, Qingdao 266061, China

**Keywords:** non-grain production, multifunctional assessment, cultivated land protection, differentiated control measures, rural revitalization

## Abstract

The control of non-grain production (NGP) has become a great challenge for cultivated land protection in China in recent years. A control method for NGP that can coordinate the conflicts between cultivated land protection and farmers’ interest is urgently needed. Taking Tongxiang City as an example, this research proposed a solution for the control and management of NGP based on cultivated land multifunctional assessment. The GIS and AHP approach were used to assess production function via a comprehensive evaluation index. The InVEST and FMSPA models were applied to assess ecological function while, the Maxent model was applied to assess recreational function, then multifunctional comprehensive zoning was conducted through natural breakpoint method and spatial overlay analysis. Five development-oriented function zones were considered, including the core area of grain production plus areas for ecological agriculture, leisure agriculture, compound agriculture, and general farmland. Differentiated control measures for NGPs in each functional subarea are proposed considering the current NGP distribution of Tongxiang city. This research can provide a reference for subsequent improvement of land management policies and can aid the achievement of sustainable agricultural development and rural revitalization.

## 1. Introduction

Cultivated land is the foundation for economic development and social stability and is a key factor in regional food and ecological security [1,2]. As a populous country with scarce land per capita, the issues of cultivated land protection and agricultural development have always been crucial concerns in China. Over the past few decades, China has established a relatively complete system of farmland protection policies, which has effectively reduced the large-scale loss of farmland triggered by urban expansion [3,4]. However, with changing dietary preferences and the mounting demand for diversified agricultural products, the protection of cultivated land has confronted a new daunting problem called NGP.

NGP is defined as a shift in production within cultivated land from a structure dominated by grain crops to one reflecting the increased production of higher-value horticultural and animal products (livestock and aquaculture) [5]. This phenomenon has increased significantly in recent years under the catalyst of land transfer and agricultural industrial policy [6,7]. A field survey by Kong (2020) combined with statistical data has preliminarily determined that the rate of NGP in China is currently approximately 27% [1], although the specific type and degree of NGP in various areas across the country shows regional disparities [5]. Non-grain expansion leads to changes in cultivated land use patterns, and quantities and spatial distributions, rendering threats to food production, biological diversity and regional ecology and the environment [8]. However, at the same time, NGPs could bring wealth for farmers and prevent further widening of the rural-urban income gap [5,9]. Therefore, how to control the moderate development of NGP to achieve the balance between grain security and the increase of farmers’ income has important practical significance [10]. However, contemporary studies taking NGP as the subject have primarily focused on its temporal and spatial distributions [5,6], as well as the driving forces of different NGP types [7,11]. Others have focused on the impact of land and agricultural industrial policies on non-grain expansion [9] or on estimating the environmental consequence of NGP [8]. There are only a few studies on the control and management of NGPs. (1) For the study of scale and region, studies are usually discussed at the macrolevel based on summarized cross-regional sample survey data, ignoring the fact that the extent and type of NGP and the expansion mechanism have conspicuous regional disparities due to differences in natural and social conditions [6]. (2) Regarding research method, most studies are qualitative analyses of policy and measures [12], and this issue has barely been quantitatively analyzed. (3) Regarding the criterion of control and management, previous studies have mostly favored a “one size fits all” approach, that is, prohibiting NGP [12]. Since farmers are endowed with autonomy in land use under the household responsibility system, such a policy is difficult to implement in actual grassroots work [7,13]. Furthermore, those studies have generally considered NGPs as a whole [6] and differentiated management and control measures for different NGP types have been little researched. Whether from the perspective, contents, or the criterion of NGP control and management, most studies have ignored the huge disparities between different NGP types and NGP development in different regions and lacked consideration for the benefit claim of farmers.

The conversion to NGP is an economically rational adjustment in accordance with comparative advantages and market demand. As cities and villages are becoming tightly connected [14], city people are becoming potential consumers of the village, and their demands for agriculture experiencing and rural landscape sightseeing are continuously increasing [15]. By fully tapping the multifunctional value of cultivated land, transforming cultivated land into an effective carrier to meet the multiple needs of agricultural industrial structure adjustment, farmers’ income increase, and urban residents’ rural tourism, will effectively solve the current dilemma of the non-grain phenomenon. The multifunctional theory of cultivated land has been widely recognized and utilized in the optimization of cultivated land use by scholars and policymakers [15], with studies covering the trade-offs among multiple functions [16,17], patterns of spatiotemporal change [18], and cultivated land management [19]. During the promotion of rural revitalization, multiple functions for cultivated land can result in achieving social equity, economic growth, and environmental sustainability.

Taking Tongxiang City as an example, this study aims to propose a solution to control non-grain expansion based on a cultivated land multifunctional assessment. To that end, the specific goals of this article are: (1) to evaluate cultivated land from the perspective of production function, ecological function, and recreational function based on multifunctional theory; (2) to delineate different cultivated land function zones in light of development orientation and aforementioned evaluation results, and (3) to propose differentiated NGP guidance and management measures for different cultivated land zones. Our analysis will provide a viable solution for the rational use of cultivated land resources in the context of rural revitalization.

## 2. Materials and Methods

### 2.1. Study Area

**Figure 1:**
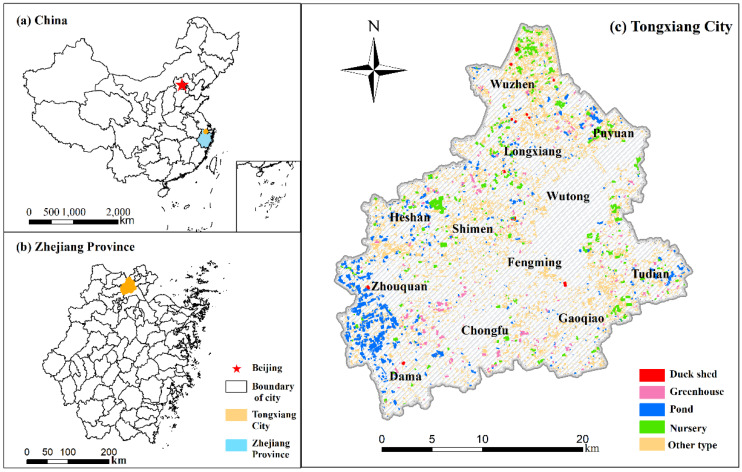
Location of the study area: (**a**) The location of Tongxiang City in China. (**b**) The location of Tongxiang City in Zhejiang Province. (**c**) The development of NGP in Tongxiang City in 2019.

This research was conducted in Tongxiang City (120°17′40″–120°39′45″ E, 30°28′18″–30°47′48″ N), which is located in the central part of the Hang-Jia-Hu Plain (Figure 1). Dominated by a subtropical monsoonal climate, the Tongxiang region has abundant sunshine and sufficient rainfall, with an annual mean temperature of 17.5 °C and an annual rainfall of 1193 mm. This district is richly endowed with cultivated land resources and water resources. Given its favourable farming conditions, Tongxiang has served as an essential commodity grain base in southeastern coastal China since time immemorial. However, Tongxiang has witnessed significant NGP expansion in recent years, with the area dedicated to NGP increasing by 4773.78 hectares during 2000 and 2018. In 2018, NGP accounted for 12.4% of the total cultivated land area. As it exemplifies non-grain development in the southeast coastal area of China, Tongxiang was selected to explore management and control solutions of NGP.

### 2.2. Data Source and Processing

Various datasets were used in this research, including land use surveys and planning data, remotely sensed images, and open data from the internet. Detailed descriptions are shown in Table 1.

### 2.3. Research Methodology

**Figure 2:**
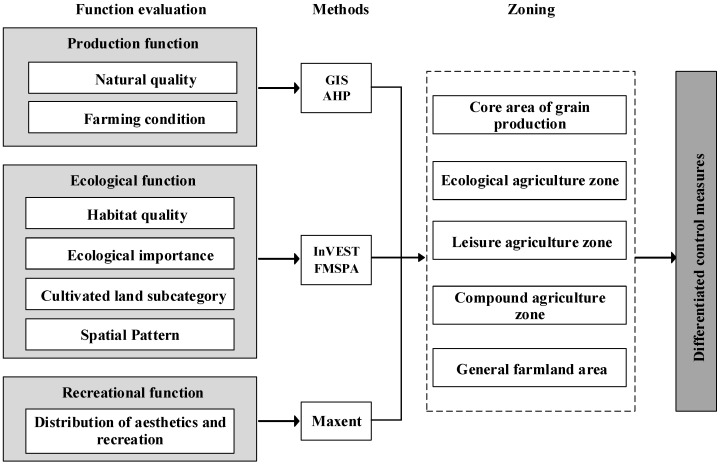
Flowchart of the datasets and approaches employed in the evaluation and zoning of the cultivated land in this study.

The research framework for delineating the different zones of cultivated land for NGP in the Tongxiang region is described below (Figure 2) (1) Multiple models and indicators were used to evaluate the cultivated land from the perspective of production, ecological, and recreational functions. (2) Five different zones were delineated based on the spatial overlay and connectivity analyses. (3) Differentiated measures for controlling and managing NGP were proposed for each zone. Details of the evaluation of different functions follow.

#### 2.3.1. Evaluation of Production Function

Production function is the most basic guarantee offered by cultivated land [20]. Ensuring national grain production and safety is the basic premise and the bottom line driving the moderate development of NGPs. According to previous studies, scholars have widely applied methods to establish a comprehensive evaluation index system due to the simple and quick data acquisition and calculation [21].

**Figure 3:**
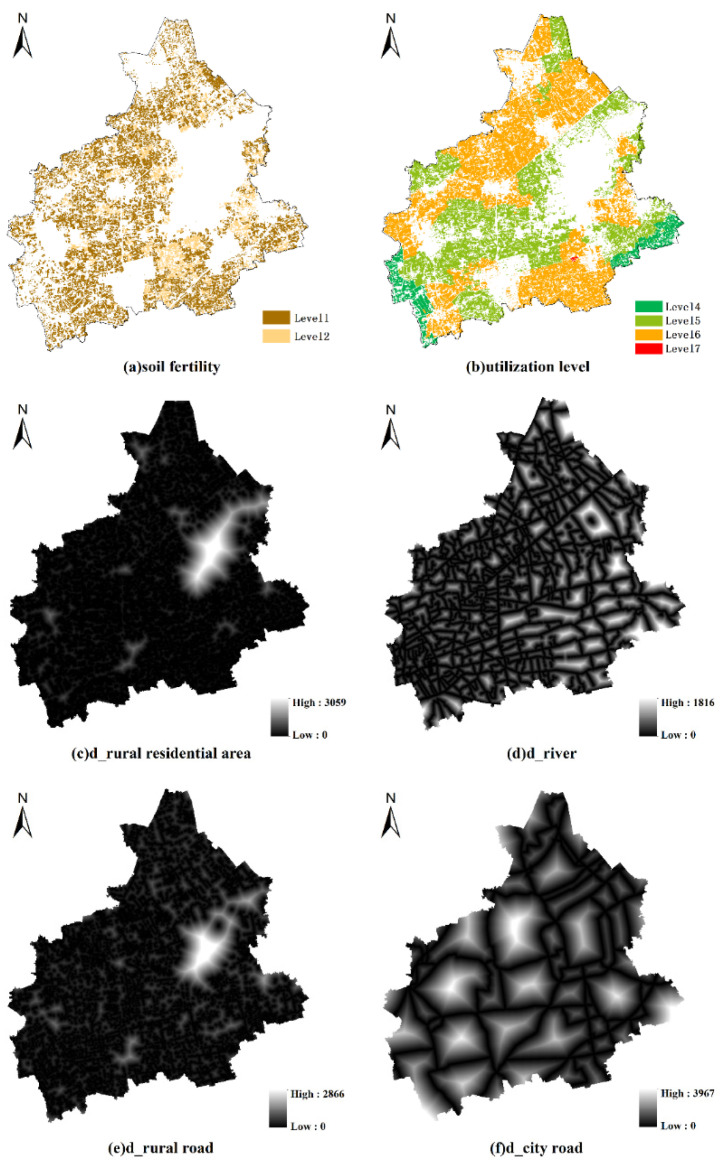
Map of cultivated land condition factors in Tongxiang city: (**a**) soil fertility; (**b**) utilization level of cultivated land; (**c**) distance to rural residential area; (**d**) distance to river; (**e**) distance to rural road; (**f**) distance to city road.

This study evaluated the production function of cultivated land considering two aspects, natural quality and cultivated conditions, for which six indicators were selected (Table 2). The spatial distribution of those factors is shown in Figure 3. The analytic hierarchy process (AHP) method was used to determine the weight of each evaluation index. The AHP builds a pairwise judgement matrix to capture the importance of the evaluation indicators and quantify the decision-makers’ judgements, ranks all evaluation indicators according to their importance, and applies the square root to calculate the weight of each factor [22]. Each evaluation index was scored quantitatively, and a dimensionless score of [0–100] was assigned (Table 3). The weighted sum method was used to calculate the final score of the production function of each parcel of cultivated land. The calculation formula is as follows:

(1) $$Y_{j}=\sum_{i=1}^{n}w_{i}\times f_{ij}$$

where *Y_j_* represents the comprehensive score for the productivity of cultivated land patch *j*, *n* is the total number of indicators, and *w_i_* and *f_ij_* are the weight and dimensionless score of index *i*, respectively.

#### 2.3.2. Evaluation of Ecological Function

**Figure 4:**
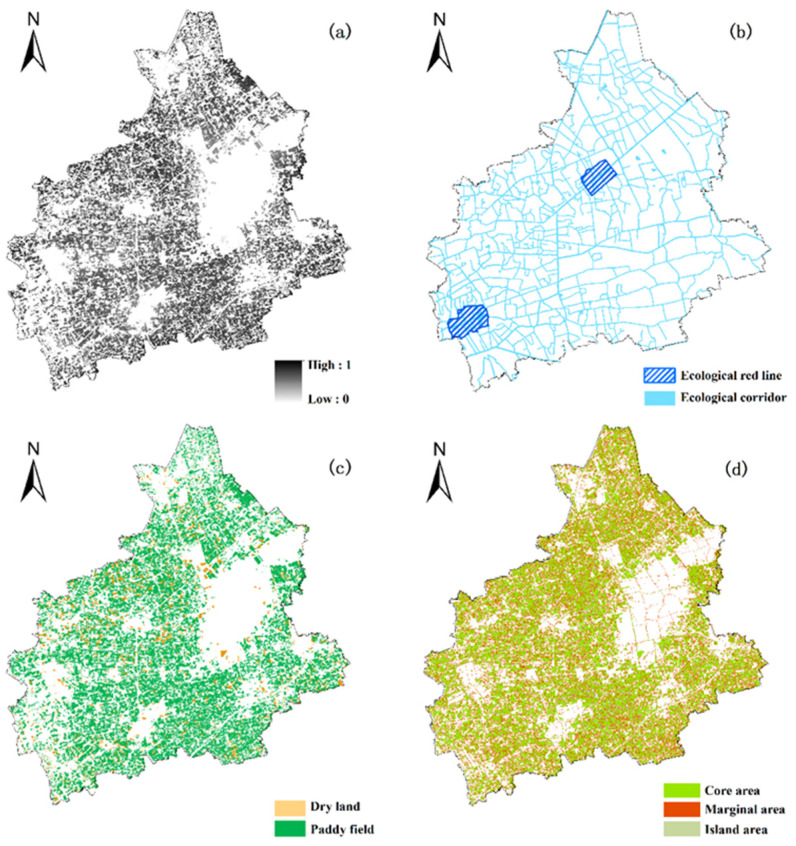
Ecological function evaluation factors of cultivated land in Tongxiang City: (**a**) habitat quality; (**b**) ecological importance; (**c**) cultivated land subcategory; (**d**) spatial pattern of cultivated land.

Cultivated land serves important ecological functions, such as water and soil conservation and biodiversity protection. To ensure the sustainable use of cultivated land, it is vitally important to use it to maintain a good ecological environment while also preventing its excessive use and destruction [23]. Based on data availability, our research spatially described the capacity of cultivated land to provide ecological services through a comprehensive index system referenced to previous studies. Four factors were selected to capture the importance and stability of cultivated land ecosystems: habitat quality, ecological importance, cultivated land subcategory, and spatial pattern (Figure 4).

(a) Habitat Quality

The habitat maintenance function of cultivated land refers to the potential of cultivated land ecosystems to provide species with suitable conditions for survival, reproduction and development; to some extent, they can reflect regional biodiversity [24]. The InVEST model has been widely used in ecosystem function evaluation due to its strong spatial analysis capabilities, high evaluation accuracy, and convenient data input [25,26]. The habitat quality module of the InVEST model evaluates the quality of a system’s habitat considering habitat suitability and the threats posed by external factors (Table 4). Given that construction land is an area with a dense distribution of human beings, it threatens the quality of regional habitats to varying degrees. This paper treats all construction land, including urban land, rural settlements, transportation land and other construction land, as a source of threats. As the intensity of human activity decreases, the intensity of the threat also declines. The spatial attenuation distance was set to 0.5–2 km according to the spatial influence range of different land use types (Table 5). The habitat types, habitat suitability and threat sources were imported into the InVEST model, and a spatial distribution map of cultivated land habitat maintenance was produced. The habitat maintenance index ranges from 0 to 1, where a value of 1 indicates that the cultivated land provides the strongest habitat maintenance function, whereas 0 suggests it provides the weakest. The formula for calculating the habitat quality maintenance index is as follows:

(2) $$Q_{j}=H_{j}\;(1-(\frac{D_{j}^{z}}{D_{j}^{z}+k^{z}}))$$

where *Q_j_* is the habitat quality of habitat type *j*, *H_j_* is the habitat suitability of habitat type *j*, *k* is the half-saturation constant, and its value is equal to the value of *D* when 1 − ($\frac{D_{j}^{z}}{D_{j}^{z}+k^{z}}$) = 0, *z* is a normalized constant equal to 2.5, *D_j_* is the habitat threat level for habitat type *j*, and its calculation formula is as follows:

(3) $$D_{\mathrm{xj}}=\sum_{r=1}^{R}\sum_{y=1}^{Y_{r}}\left(\frac{W_{r}}{\sum_{r=1}^{R}W_{r}}\right)r_{y}i_{\mathrm{rxy}}\beta_{x}S_{\mathrm{jr}}$$

where *D_xj_* is the habitat threat level of grid *x* in habitat type *j*, *W_r_* is the weight of the threat source and indicates the relative destructive power of each threat to the habitat, *R* is the type of threat source, *β_x_* is the accessibility level of grid *x* with a value ranging from 0 to 1, in which the higher the *β_x_* value, the easier grid *x* is to reach, and *S_jr_* is the sensitivity of habitat type *j* to the threat source *r* which has a value ranging from 0 to 1.

(b) Ecological Importance

The ecological red line refers to areas that are designated to mitigate eco-environmental problems, protect the integrity and connectivity of ecosystems, and balance the trade-offs between eco-environmental protection, resource utilization, and economic development [27]. The ecological red line plays an essential role in guaranteeing regional ecological security and sustainable development. Ecological corridors such as rivers are also important for regulating hydrological systems and protecting biodiversity. Therefore, cultivated land is divided into ecological red lines, ecological corridors, and other areas based on the importance of ecological protection and assigned values of 100, 50, and 20, respectively (Table 6).

(c) Cultivated Land Subcategory

Previous studies on the value of cultivated land ecosystem services divided cultivated land into three categories: paddy fields, dry land and irrigated land. The total values of the ecosystem services of the three types are 7562.8 yuan/ha, 6114.3 yuan/ha, and 7973.4 yuan/ha, respectively [28]. Tongxiang city has only two types of cultivated land, paddy fields and dry land, which are assigned scores of 100 and 80, respectively.

(d) Spatial Pattern of Cultivated Land

Contiguous cultivated land better maintains the material and energy exchange between cultivated land patches, protects animal habitat and biodiversity, and ensures the stability of cultivated land ecosystems [29]. This research used farmland morphological spatial pattern analysis (FMSPA), proposed by Cheng et al. (2017), to identify and characterize different spatial forms of cultivated land [20]. Using mathematical morphology imaging processing techniques, combined with the law of distance decay in geography and edge effects in landscape ecology, this method can automatically identify and classify the pixel types in binary images through a series of mathematical operations such as dilation, corrosion, opening and closing [30]. Considering the mechanism through which non-agricultural land interferes with cultivated land ecosystems, the model first converts cultivated land patches into binary raster data and then performs erosion and expansion operations to divide the cultivated land ecosystem into matrix farmland, edge farmland and island farmland. Matrix farmland refers to the inner contiguous area of the cultivated landscape, which is a potential, suitable and complete living environment for creatures. Marginal farmland refers to the junction between the outside of the matrix farmland and other land types, and provides a buffer for the matrix farmland. Island farmland refers to isolated, small arable land patches surrounded by other land types with low ecosystem stability that are easily affected by surrounding land types. For the FMSPA, first, data binarization was conducted. The cultivated land map is converted to binary grid data with a resolution of 10 m, where cultivated land is assigned the value 2 (foreground), and non-cultivated land is assigned the value 1 (background). Then, the model is run, and the matrix farmland, edge farmland and island farmland are identified in turn.

#### 2.3.3. Evaluation of Recreational Function

Cultivated land with beautiful rural scenery and a profound farming culture delivers recreational services by providing leisure opportunities and entertainment for residents and tourists [31]. With the increasing demand to meet spiritual needs, the value of cultivated land in the aesthetic landscape and in the inheritance of local culture has become increasingly valued [19]. A methodology is urgently needed that embraces the principle of rational cultivated land utilization informed by the wider evaluation of recreational services.

POI (Point of interest) has been widely used in research in geography, ecology, sociology and other fields due to its broad coverage, rich types, large number, precise spatial location and information on people’s preferences [32]. This article collected the POIs with the “agritainment”, “leisure farm” and “scenic spot” labels for the recreational function evaluation, and these were input into the Maxent model as the occurrence localities of given observation points.

Using a machine learning technique, the Maxent model can map the potential distribution of an object by exploring the relationships between a set of environmental grids and the occurrence of localities of given observation points [15]. Since it requires only presence data, and shows good performance even with small sample sizes, this study adopted the model to simulate and evaluate the spatial distribution of farmland leisure aesthetic functions. Two sets of environmental variables, natural variables and human variables, were selected based on their relationships with farmland leisure aesthetics (Table 7), as demonstrated in previous studies [33,34]. All variables were transferred to the WGS_1984_UTM_Zone_50N projection system and ASCII raster grid pattern before the model run.

## 3. Results

### 3.1. Evaluation of Production Function

**Figure 5:**
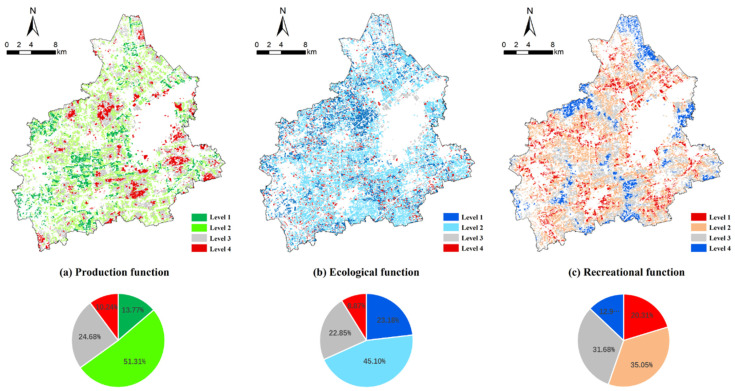
Evaluation of different functions of Tongxiang City; (**a**–**c**) represent production function, ecological function, and recreational function, respectively.

The evaluation results showed that the score of the cultivated land production function in Tongxiang city was between 43.07 and 95.66. The natural breakpoint method was used to divide all the land patches into four levels. Among them, the land parcels with a score between 92.71 and 95.66 were classified as the first level. With a total area of 4015.91 hectares, the first level represents the most fertile land with the highest production function and accounts for 13.77% of the total cultivated land. Moreover, the land patches with scores of 89.22 to 92.71, 86.33 to 89.22, 43.07 to 86.33 were classified as the second level, third level, and fourth level, respectively, and accounted for 51.31%, 24.68%, and 10.24% of the total cultivated land (Figure 5). The first level was mostly distributed in the contiguous cultivated land of the towns of Fengming and Chongfu, while the fourth level land was concentrated in areas close to construction land in Longxiang and Wutong town.

### 3.2. Evaluation of Ecological Function

The scores of different indicators were quantified separately, and the weights were given through the AHP (Table 6) to build a comprehensive evaluation index system for the ecological function of cultivated land.

The evaluation results show that the ecological function score of cultivated land in Tongxiang city was between 37.38 and 99.99 (Figure 5). The natural breakpoint method was adopted to divide all the land patches into four levels. The land parcels with a comprehensive score between 77.54 and 99.99 are classified as the first level, had a total area of 6761.68 hectares, and account for 23.18% of the total cultivated land. The land patches with scores of 66.71 to 77.54, 54.14 to 66.71, and 37.38 to 54.14 were classified as the second level, third level, and fourth level, respectively, and accounted for 45.10%, 22.85%, and 8.87% of the total cultivated land. The land patches with the strongest ecological function are concentrated in Longxiang and Wutong town, while those with the lowest ecological function are most often marginal land and are distributed sporadically.

### 3.3. Evaluation of Recreational Function

The recreational function of cultivated land evaluated by the Maxent model is shown in Figure 5. The natural breakpoint method was adopted to divide all the land patches into four levels. The land parcels with output scores between 0.671 and 0.994 were classified as the first level, have a total area of 5925.05 hectares, and account for 20.31% of the total cultivated land. The land patches with scores of 0.431 to 0.671, 0.187 to 0.431, and 0 to 0.187 were classified as the second level, third level, and fourth level, respectively, and accounted for 35.05%, 31.68%, and 12.96% of the total cultivated land, respectively (Figure 5). The land patches offering a high supply of recreational functions are concentrated in the ambient areas of cities and towns, while those with low recreational functions are distributed in the most remote areas.

### 3.4. Multifunctional Comprehensive Zoning

**Figure 6:**
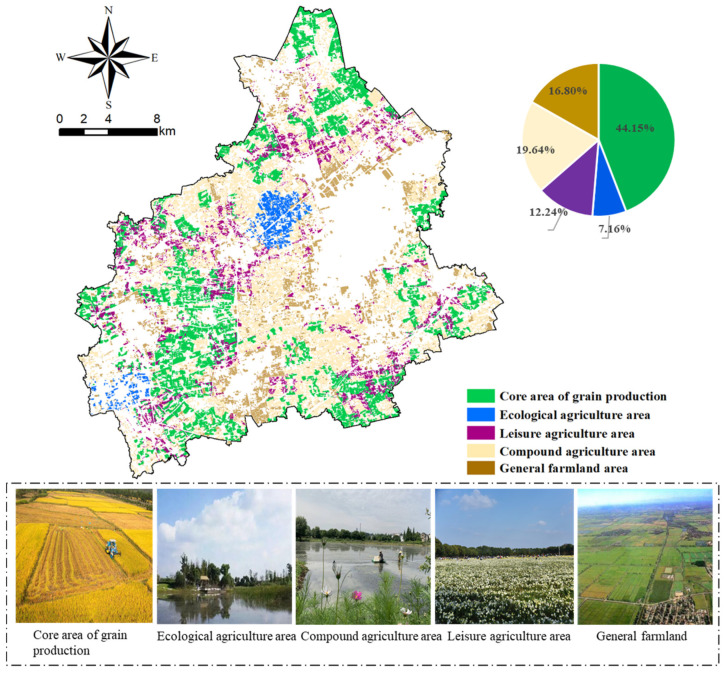
Multifunctional comprehensive zoning of cultivated land in Tongxiang City.

Multifunctional comprehensive zoning was conducted based on natural breakpoint method and spatial overlay analysis through GIS. According to the evaluation results of multi-functions, natural breakpoint method was used to divide all the land patches into several levels. The first-level areas of production, ecological and recreational function evaluation were regarded as the core area of grain production, ecological agriculture area, and leisure agriculture area respectively. The remaining areas within the basic farmland were delimited as compound agriculture area, and those outside the basic farmland were delimited as general farmland areas. The core area of grain production is the largest zone, with an area of 12,878.51 hectares, and accounts for 44.15% of the total cultivated land area (Figure 6). The other zones, including ecological agriculture areas, leisure agriculture areas, compound agriculture areas, and general farmland areas, account for 2089.74, 3571.67, 5729.13, and 4899.53 hectares, respectively.

Different functional areas exhibited large gaps in spatial distribution except for the quantity structures. (1) Core area of grain production: This area is the most widely distributed zone within all cultivated land, and the most continuous area of land was located in the west and south areas. Characterized by fertile soil, concentrated and contiguous plots, and outstanding productivity, this zone holds the important task of enhancing grain supply capacity and guaranteeing grain production. (2) Ecological agriculture area: This area was mainly distributed in the centre and southwest, and the distribution scale was small. (3) Leisure agriculture area: This area integrated agricultural production and residents’ relaxation, and it was mainly located around the urban areas in the centre and northeast. The areas in the west and southeast were connected to the core area of grain production. (4) Compound agriculture area: This area was the second largest zone and had a widespread distribution. By integrating grain production and NGP, this area could alleviate contradictions between environmental benefits and economic profits. (5) General farmland area: This area was mainly concentrated in marginal areas of cultivated land as well as in land parcels around construction land.

### 3.5. Results of Spatial Overlay Analysis

**Figure 7:**
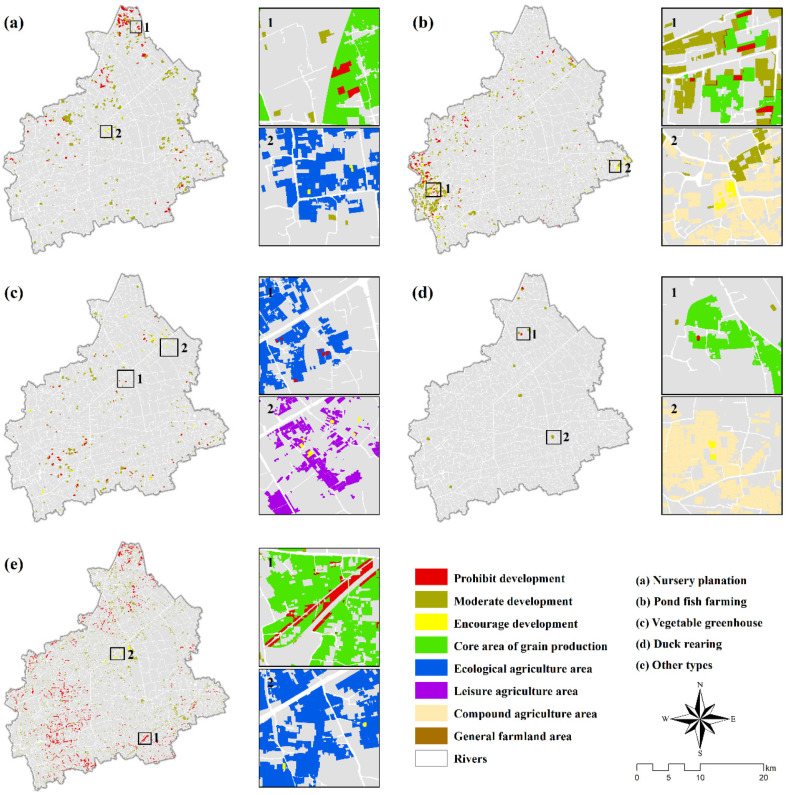
Spatial overlay between the current NGP distribution of Tongxiang city and multifunctional comprehensive zoning. (**a**). Nusery planation. (**b**). Pond fish farming. (**c**). Vegetable greenhouse. (**d**). duck rearing. (**e**). other types.

An overlay analysis was carried out between the current NGP distribution of Tongxiang city and the abovementioned multifunctional comprehensive zoning (Figure 7). Three levels of future control measures for current NGP types were identified, including prohibitive development, moderate development, and encouraged development.

The prohibitive development region of nursery plantations was mainly concentrated in the northern part of Tongxiang, Wuzhen town, where there is plenty a large core area of grain production. Since all types of NGPs should be prohibited in this zone, the land parcels that have been converted to nursery plantations must be restored to grain production in the future. In contrast, nursery plantations were encouraged to develop in ecological agriculture areas (Figure 7a). Pond fish farming is the most numerous NGP type in Tongxiang city and is mainly distributed in the southwestern part of Zhouquan town and Dama town. Pond fish farming was encouraged to develop in compound agriculture areas but prohibited in the core area of grain production and ecological agriculture areas (Figure 7b), which is the same for duck rearing (Figure 7d). Vegetable greenhouses were prohibited from developing in the core area of grain production and ecological agriculture area but encouraged in the leisure agriculture area (Figure 7c). Other types mainly refer to unused land within cultivated land and land parcels used for roadside greening. Most of these land parcels could be restored to grain production in the future.

## 4. Discussion

### 4.1. Exploration of the Control and Management Measures for NGP Development

Since the General Office of the State Council issued the Opinions on Preventing Non-grain Production within Cultivated Land and Stabilizing Grain Production (No.44 [2020]) in 2020, the control and management of NGP has become a hot area of focus within the study of cultivated land protection and utilization [7,9,36]. Optimization is the basis of control, and control is the realization of optimization [37]. On the one hand, non-grain expansion must be controlled to guarantee national grain security while simultaneously reducing a negative impact on the ecological environment [8]. On the other hand, the multifunctional value of cultivated land should be fully exploited, and the relationships between different functional zones should be coordinated [15,17].

This study considered the local resources, environment and agricultural development of the study area and explored how to balance the interests of farmers and country, as well as how to realize the rational use of cultivated land resources against the background of rural revitalization [10]. Considering the huge disparities of different types of NGPs in their economic benefits, environmental impact, and overall sustainability demonstrated in our previous studies [5,8], this study divided cultivated land into five development-oriented zones controlled at three levels and proposed differentiated guidance and control measures for future NGP development.

The first level of control corresponds to the core area of grain production. The prerequisite for the rational development of NGPs is that national grain security and cultivated land productivity must be guaranteed. In this zone, high-quality and contiguous farmland ensures minimum non-agricultural interference and maximum grain production [20]. All types of NGPs should be prohibited in this zone, and land parcels that have been converted to NGPs must be restored within a limited period. Land parcels with a high probability of NGP conversion could be monitored by remote sensing in the future [7]. Since land resource utilization and health largely depend on how smallholders perform [38], linking grain production to subsidies and rewards would boost farmers’ enthusiasm. Moreover, incorporating these issues into the assessment system of local government officials could also guarantee grain production and grain security [39].

The second level of control corresponds to the ecological agriculture area, leisure agriculture area, and compound agriculture area. Combined with grain production, certain NGP types should be encouraged to develop in these areas to satisfy the increasing demand of inhabitants for diversified food, natural spaces and cultural experiences [40]. Specifically, ecological agriculture areas should enhance the ecological quality of farmland and facilitate sustainable agricultural development. Only NGP types with high environmental sustainability, such as nursery plantations, should be allowed, while existing NGP types with severe adverse impacts on the ecological environment should be prohibited and phased out, such as duck rearing [37]. As agriculture is gradually becoming a path for maintaining varied landscapes via regionally differentiated farming systems, it has high rural tourism potential [40]. The aim of leisure agriculture areas is to promote agricultural culture conservation while generating economic benefits with the support of the landscape and the preservation of local culture. Farmland landscape sightseeing and vegetable or fruit picking could be developed in this area. Moreover, some existing fish ponds that are difficult to dismantle could be transformed into mulberry fishponds, one of the globally important agricultural heritage systems, which can provide unique agricultural cultural tourism opportunities that integrate ecological sightseeing, leisure experiences and science popularization [41]. Compound agricultural areas encourage compound agricultural models such as fish-rice farming and duck-rice farming, which can establish a benign material recycling system, increase the yield and benefit of cultivated land per unit area, and reduce environmental pollution [42]. The specific production types are selected based on geographical location, resource endowment and traditional culture, fully revealing the distinctive regional characteristics. Related standards, such as those for the ditches used in fish and shrimp breeding, should be set to reduce the negative impact on the productivity of cultivated land.

The third level of control corresponds to the general farmland area aside from basic farmland. This zone aims at achieving the maximum economic benefits from land resources and implements relatively loose management measures. Various NGP types can be appropriately developed in this zone on the premise of avoiding damaging the plough layer or diminishing cultivated land productivity.

### 4.2. NGP Development and Rural Revitalization

In the context of rural revitalization, land-use policies related to post-productivism is increasing in China [43,44]. Post-productivism emphasizes high value-added components and innovation in the rural economy and believes that rural production should be diverse rather than specialized in grain production [45]. Located in the economically developed Yangtze River Delta region, the demand for diversified food, agricultural experiences and rural landscape sightseeing in Tongxiang city are continuously increasing among modern affluent urban inhabitants, which provides the impetus for the multiple utilization of cultivated land resources. Therefore, we believe that it is more suitable to implement policies of post-productivism to meet the needs of urban residents’ consumption and promote the diversification of land use when exploring management and control measures for NGPs, rather than policies of productivism that only guarantee the safety of food production.

China is still in the process of rapid urbanization, and the issues of population loss and economic downturn are severe in rural areas. The key to rural revitalization is to explore the rural value and promote the income growth of farmers to change the passive role of rural areas in population and capital resource competition with urban areas. Proper land use planning and policies play a vital role in guiding rural transformation in China. Huang et al. (2020) took Yuanqianshe in Xiamen, China, as an example and demonstrated the success of a suitable policy to help villages move towards revitalization [44]. The Yuanqianshe villagers promoted land-use optimization through place-making, including traditional productive farmlands changing into urban vegetable fields, kinds of orchards, and DIY agricultural activities. This is similar to the findings of this study, which will reconstruct the economic interest ties of villagers and help revitalize the village.

Furthermore, a requirement of rural revitalization is that enhancing rural economic development should be based on the premises of ecological preservation and environmental improvement. While economic profit is always the focus of farmers’ decision making [46], policy intervention and interest adjustment by the local government are necessary to guide the direction of agricultural development. Our control and management measures for NGP development are proposed based on the environmental benefits and comprehensive sustainability of each NGP type [5,8], which is conducive to alleviating environmental pollution and promoting comprehensive benefits. With regard to those land parcels that converted to NGP spatial clusters and were too difficult to be dismantled, moderate environmental taxes could be imposed based on their negative impact.

### 4.3. Limitations and Research Prospective

Agriculture and land policy should aim to integrate an understanding of natural science within a socioeconomic framework that addresses issues of governance, equity, and coordination. In practice, it requires an appropriate science-to-policy interface with multilevel governance and interactions between scientists and farmers [47]. This study delineated various development zones for cultivated land and proposed differential control and management measures of NGPs in each zone, which quantified the recommendations of previous scholars [44]. These countermeasures can effectively stabilize food production and firmly secure the lifeline of national food security. Concurrently, the method fully took the farmers interests into consideration and allowed the proper development of NGPs, which is conducive to achieving the realization of rural revitalization and common prosperity. While this study proposed a new concept and an effective methodology for the guidance and control of NGPs, some limitations still exist in the study, and some suggestions for further study are proposed.

First, there are currently no common principles or guidelines to help select the most representative indicators for a multifunctional evaluation of cultivated land [48]. It is also difficult to fully take the various indicators into consideration due to data availability. The indexes can be enriched by collecting more field survey data and detailed statistical data through further studies. Second, although the multiple functions of cultivated land were evaluated, the interactive relationship between different functions was not investigated. Therefore, in future studies, it will be necessary to incorporate the trade-offs/synergies of different functions into the boundary delimitation between various development zones to provide more information to improve the coordinated development of different zones. Furthermore, PPGIS can be adopted to bring more stakeholders’ place-based knowledge and values to support better land use management [49].

## 5. Conclusions

A quantitative method for the guidance and control of NGP is key to optimizing cultivated land utilization and enhancing cultivated land protection. Taking Tongxiang city as an example, this study employed a comprehensive evaluation of cultivated land from the “production-ecology-recreation” perspective. Five different function zones of cultivated land were delimited combined with the current arable land protection layout: the core area of grain production, ecological agriculture area, leisure agriculture area, compound agriculture area, and general farmland area. Five function zones are controlled at three levels and differentiated guidance and control countermeasures for NGP development are proposed. While the core area of grain production prohibits all types of NGPs, other zones could develop certain NGP types appropriately on the premise of ensuring grain security and protecting cultivated land production capacity. This new idea avoids the “one size fits all” solution of previous management and control measures for NGPs and can help the agricultural areas of China strictly adhere to the cultivated land protection red line and the bottom line of grain production.

## Figures and Tables

**Table 1:** Description of the data used in this paper.

Data	Resolution	Source
Land use survey data	1:10,000	Land and Resources Bureau of Tongxiang
Aerial photographs	0.5 m	Land and Resources Bureau of Tongxiang
Google Earth images	0.13 m	Google Earth
Road network	1:10,000	Extracted from the land use survey data
Rural residential area	1:10,000	Extracted from the land use survey data
Rivers	1:10,000	Extracted from the land use survey data
Soil fertility data	1:10,000	Land and Resources Bureau of Tongxiang
Point of interest	91 points	Open platform of the Gaode map

**Table 2:** Index system and weight of comprehensive evaluation of cultivated land production function in Tongxiang.

Criterion Layer (Weight)	Index Level	Weight	Combined Weight
Natural quality (0.6250)	Soil fertility (SF)	0.5375	0.3359
	The utilization level of cultivated land (ULCL)	0.4625	0.2891
Farming condition (0.3750)	The distance to river (D_r)	0.2690	0.1009
	The distance to rural residential area (D_rra)	0.3184	0.1194
	The distance to rural road (D_rr)	0.2216	0.0831
	The distance to city road (D_cr)	0.1910	0.0716

**Table 3:** Quantitative standard for evaluation index of cultivated land production function in Tongxiang City.

Score	SF	ULCL	D_rra (km)	D_r (km)	D_rr (km)	D_cr (km)
100	1	1	≤0.5	≤0.2	≤0.2	≤1
95		2				
90	2	3				
85		4				
80	3	5			0.2~0.5	1~1.5
75		6	0.5~1	0.2~0.5		
70	4	7				
65		8				
60	5	9			0.5~1	1.5~2
55		10				
50	6	11	1~1.5	0.5~1		
45						
40	7	12				
35					1~1.5	2~3
30	8	13				
25						
20			>1.5	>1		
15	9	14				
10					>1.5	>3
5	10	15				

**Table 4:** Different cultivated land habitat types and their sensitivity to threat factors.

Type Code	Habitat Types	Habitat Suitability	Urban Land	Rural Residential Area	Traffic Land	Other Construction Land
1	Paddy filed	0.9	0.6	0.5	0.2	0.3
2	Dry land	0.8	0.8	0.6	0.3	0.5
3	Uncultivated area	0	0	0	0	0

**Table 5:** Quantitative table of habitat threat sources.

Threat Source	Maximum Impact Distance (km)	Weight	Linear Correlation of Decline
Urban land	1.6	0.9	exponent
Rural residential area	1	0.8	exponent
Other construction land	0.5	0.5	exponent
Traffic land	2	1	linear

**Table 6:** Quantitative standard of the comprehensive evaluation index of cultivated land ecological function in Tongxiang city.

Score	Habitat Quality	Ecological Importance	Cultivated Land Subcategory	Spatial Pattern of Cultivated Land
100	≥0.85	Ecological red line	Paddy field	Core area
90				
80			Dry land	
70	0.80~0.85			Marginal area
60				
50		Ecological corridor		
40				
30	≤0.80			Island area
20		Other areas		
10				
0				
Combined weight	0.2217	0.3612	0.2198	0.1973

**Table 7:** Description of the variables utilized for mapping the spatial distribution of the farmland recreational function.

Type	Variable	Calculation
Natural	Shape of cultivated land patches	Based on the perimeter and area of the farmland patches
	Cultivated land contiguity	Conefor Inputs for ArcGIS 10.x [35].
	Distance to the water	Euclidean distance [34].
Human	Distance to the town center	Euclidean distance [34].
	Distance to the village	
	Distance to the scenic spot	
	Travel time	Cost distance [33].

## Data Availability

Not applicable.
